# Analgesic and Antidepressant Effects of the Clinical Glutamate Modulators Acetyl-L-Carnitine and Ketamine

**DOI:** 10.3389/fnins.2021.584649

**Published:** 2021-05-11

**Authors:** Ulderico Freo, Viola Brugnatelli, Fabio Turco, Gastone Zanette

**Affiliations:** ^1^Section of Anesthesiology and Intensive Care, Department of Medicine-DIMED, University of Padua, Padua, Italy; ^2^Section of Dentistry, Department of Neurosciences-DNS, University of Padua, Padua, Italy; ^3^Molecular Biology and Biochemistry Laboratory, Department of Neurogastroenterology, University of Naples Federico II, Naples, Italy

**Keywords:** acetyl-L-carnitine, ketamine, regional cerebral metabolic rates for glucose, pain, depression

## Abstract

Pain and depression are leading causes of disability and of profound social and economic burden. Their impact is aggravated by their chronicity and comorbidity and the insufficient efficacy of current treatments. Morphological and functional metabolism studies link chronic pain and depressive disorders to dysfunctional neuroplastic changes in fronto-limbic brain regions that control emotional responses to painful injuries and stressful events. Glutamate modulators are emerging new therapies targeting dysfunctional brain areas implicated in the generation and maintenance of chronic pain and depression. Here, we report the effects of two clinically approved glutamate modulators: acetyl-L-carnitine (ALCAR) and S, R(±)ketamine (KET). ALCAR is a natural neurotrophic compound currently marketed for the treatment of neuropathies. KET is the prototypical non-competitive antagonist at *N*-methyl-D-aspartate glutamate receptors and a clinically approved anesthetic. Although they differ in pharmacological profiles, ALCAR and KET both modulate aminergic and glutamatergic neurotransmissions and pain and mood. We assessed in rats the effects of ALCAR and KET on cerebral metabolic rates for glucose (rCMRglc) and assessed clinically the effects of ALCAR in chronic pain and of KET in post-operative pain. ALCAR and KET increased rCMRglc at similar degrees in prefrontal, somatosensory, and cingulate cortices, and KET increased rCMRglc at a different, much larger, degree in limbic and dopaminergic areas. While rCMRglc increases in prefrontal cortical areas have been associated with analgesic and antidepressant effects of ALCAR and KET, the marked metabolic increases KET induces in limbic and dopaminergic areas have been related to its psychotomimetic and abuse properties. In patients with chronic neuropathic pain, ALCAR (1,000 mg/day) yielded to a fast (2 weeks) improvement of mood and then of pain and quality of life. In day-surgery patients, KET improved dischargeability and satisfaction. In obese patients undergoing bariatric surgery, a single, low dose of KET (0.5 mg/kg) at induction of anesthesia determined a very fast (hours) amelioration of post-operative depression and pain and an opioid-sparing effect. These findings indicate that ALCAR and KET, two non-selective glutamate modulators, still offer viable therapeutic options in comorbid pain and depression.

## Introduction

Chronic pain and depression are leading causes of disability that are frequently encountered comorbidly in a variety of clinical conditions, sharing genetic and psychological risk factors, a relapsing-chronic course, and neurobiological features (Bair et al., 2003; Meyer et al., 2007; Kroenke et al., 2011; Global Burden of Disease Study 2013 Collaborators, 2015; D’Amato et al., 2016; Freo et al., 2019a,b). In the chronic pain patient, the presence of a major depression is associated with reduced function, poorer outcome, and expanded health-care costs; in the depressed patient, pain is a frequent presenting symptom and a predictor of treatment response (Bair et al., 2003; Meyer et al., 2007; Kroenke et al., 2011; D’Amato et al., 2016).

Because of global aging, the prevalence of pain, depression, and comorbid pain and depression is expected to increase (Molton and Terrill, 2014; Chui et al., 2015). As currently available therapies do not work for many patients, new pharmacological approaches are deemed essential. Glutamate drugs are emerging treatments for pain and depression (Henter et al., 2017; Pereira and Goudet, 2019). Newly developed, receptor-selective glutamate compounds are often hampered by the uncertain toxicity and the tolerability profile; older, non-selective agents are available for different routes of administration [i.e., oral (PO), intramuscular (IM), and intravenous (IV)] and continue to be investigated actively (Henter et al., 2017; Pereira and Goudet, 2019).

We assessed the effects of two clinical glutamate modulators, acetyl-L-carnitine (ALCAR) and ketamine (KET), on regional glucose cerebral metabolism and on patients with comorbid pain and depression. This review summarizes our pre-clinical and clinical research on ALCAR and KET.

## Pain and Depression

According to the International Association for the Study of Pain, pain can be roughly classified on the basis of mechanism as nociceptive, neuropathic, or nociplastic pain. Nociceptive pain reflects the normal functioning of the somatosensory systems responding in a stimulus-dependent manner to an actual or potential damage of non-neuronal tissue and is treated with conventional non-steroidal anti-inflammatory and/or opioid analgesics (Freynhagen et al., 2019). In contrast to nociceptive pain, neuropathic pain is induced by a lesion or disease of the somatosensory nervous system that generates and maintains spontaneous pain and positive and negative sensory disturbances, independently from stimuli (Freynhagen et al., 2019; Scholz et al., 2019). Neuropathic pain worsens cognitive and mood functions and quality of life and is treated with antiepileptic and/or antidepressant drugs targeting the abnormal somatosensory nervous systems (Nicholson and Verma, 2004; Fornasari, 2017). Recently, the International Association for the Study of Pain defined nociplastic pain as pain occurring from an altered nociception in spite of no evidence of any tissue damage (Freynhagen et al., 2019). Multiple pain mechanisms may be active at the same time in the single patient, making diagnosis and treatment more difficult (Freynhagen et al., 2019). Finally, pain is considered chronic if it lasts longer than the 3 months’ healing time.

Neuropathic pain is often chronic, and neuropathic symptoms (i.e., “component”) are frequently reported and aggravate painful, non-primary neurological conditions in spite of no demonstrable neuronal injury (Freo et al., 2019b, 2020; Freynhagen et al., 2019; Scholz et al., 2019). Because of the loss of protective features and the damage they cause, chronic and neuropathic pain are viewed as “disease states” (Costigan et al., 2009).

Although they often coexist and complicate each other’s outcome, the exact relation between chronic pain and depression has yet to be elucidated (Bair et al., 2003; Sheng et al., 2017). Most studies report an increased sensitivity to experimental pain and, therefore, a decreased pain threshold in depressed compared to non-depressed subjects especially when emotional aspects of experimental pain are taken into account (Ushinsky et al., 2013); probably because of an altered sensation, unexplained pain is common in depression and is often the presenting and prevailing symptom (Bair et al., 2003). Stressful events may facilitate chronification of both pain and of negative/depressed mood; personal experiences such emotional strain, childhood traumatic experiences and post-traumatic stress disorder, and negative social and work experiences are associated with a higher risk of developing depression and/or chronic pain; personal attitudes such as catastrophizing and low self-efficacy are also risk factors of developing either or both states (O’Sullivan, 2004; Edwards et al., 2016). In a large genetic study, different pain phenotypes presented robust and positive genetic correlations with each other as well as with depression, suggesting common underlying genetic factors between pain and depression (Meng et al., 2020). Because it has a much higher incidence, approximately tenfold, than other mental disorders, evolutionary and pain psychologists have attempted to explain pain and depression in terms of behavioral adaptiveness. In this contest, Baliki and Apkarian (2015) support that nociception is essential to protect individuals from injury not only by inducing conscious pain and active avoidance behaviors but also by modulating automatic motor behaviors continuously and in the absence of overt pain. Similarly, a negative mood may be reconceptualized as a psychic pain that may be protective against environmental dangers in complex and hierarchical societies and promote healing; even persistent pain after an injury may have an adaptational value in that it favors survival after injuries that impair motor functions and increase vulnerability (Gałecki and Talarowska, 2017). In contrast, within the evolutionary framework, chronic pain beyond normal healing and chronic or relapsing depression are viewed as maladaptive processes which are maintained by neuropathological abnormalities.

## Glutamate Drugs

### Neuroplasticity

Neuroplasticity indicates the brain’s ability to change over time and, more specifically, the ability of strengthening or weakening the synaptic signals between neurons in response to a variety of physiological stimuli such as behavioral, cognitive, and motor activities, as well as after pathological events such as painful or stressful conditions and neurological diseases (Lucassen et al., 2014; Pelletier et al., 2015). In chronic pain and depression, morphological and functional neuroplastic changes were found most pronounced in fronto-limbic regions (Mutso et al., 2012; Khan et al., 2014; Ezzati et al., 2019). The human prefrontal cortex is phylogenetically a recent brain area that matures late during development and is pivotal in the acquisition of motivational properties of different types of rewarding and aversive stimuli which include self-reference, self-appraisal, and emotion and mood control (Teffer and Semendeferi, 2012).

In chronic pain and depression, chronic exposure to stress is a common factor that may produce long-lasting changes (i.e., maladaptive neuroplasticity) in highly sensitive brain areas such as the prefrontal cortex and the hippocampus and in their functional connections, which may underlie the cognitive and behavioral impairments accompanying these conditions (Teffer and Semendeferi, 2012; Lucassen et al., 2014). For example, in comparison to healthy controls, a group of patients with chronic low back pain performing a simple visual attention task presented a reduced deactivation in regions of the default mode network; similarly, patients with a major depressive or a bipolar disorder while performing a *n*-back working memory task failed to deactivate the medial prefrontal cortex (Baliki et al., 2008; Rodríguez-Cano et al., 2017). The default mode, attention, and salience networks are all disrupted in pain and depression (Shao et al., 2018; van Ettinger-Veenstra et al., 2019). Conversely, the deep brain stimulation of the cingulate cortex relieved patients suffering from an intense neuropathic pain as well as patients with a severe treatment-resistant depression (Boccard et al., 2016; Zhang et al., 2018).

Treating pain improves depressive symptoms and *vice versa* (Skolasky et al., 2012; Rahman et al., 2020). Because the incidence of depressive and pain symptoms is increasing in parallel with the aging of the world population, it is particularly important to develop strategies that target both disorders to minimize polypharmacy and optimize therapeutic outcomes (Solhaug et al., 2012; Molton and Terrill, 2014). Although therapeutic options are available for chronic pain and depression, less than 50% of all patients treated for chronic pain report a clinically meaningful (i.e., ≥50%) pain relief with current analgesic treatments, and only about 50–60% of patients with major depressive disorders achieved remission after an adequate course with conventional antidepressants. The efficacy of treatments of comorbid pain and depression has been less studied, but it is well known that these two conditions worsen each other’s severity and therapeutic response.

Glutamate is the most abundant excitatory neurotransmitter in the central nervous system of adult mammals and has a major role in neuroplasticity (Henter et al., 2017; Pereira and Goudet, 2019). Glutamate acts through eight ionotropic and metabotropic receptor subtypes (mGluR1–mGluR8) that have been classified into three groups: Group I receptors (mGluR1 and mGluR5) are coupled to G_αq_ proteins and phospholipase C and are involved in central sensitization and pain chronification; Group II receptors (mGluR2/mGluR3) and Group III receptors (mGluR4 and mGluR6–mGluR8) are coupled to G_αq_ proteins and inhibit adenylate cyclase; their activation is effective against nociceptive and neuropathic pain (Zammataro et al., 2011; Henter et al., 2017; Pereira and Goudet, 2019).

Glutamate competitive and non-competitive ligands, binding to the same or to a different receptor site of the endogenous ligand, have been on development for at least three decades. Trials on stroke and traumatic brain injuries with competitive glutamate or glycine antagonists (i.e., selfotel, aptiganel, eliprodil, licostinel, and gavestinel) have failed (Ikonomidou and Turski, 2002). Recent trials with glutamate agents to treat pain and depression yielded promising but sometimes inconsistent results; non-selective glutamate modulators such as ALCAR and KET can still be of interest to glutamate research (Gould et al., 2019; Pereira and Goudet, 2019).

### ALCAR

Acetyl-L-carnitine (γ-trimethyl-β-acetylbutyrrobetaine) is an acetyl ester of carnitine, an endogenous molecule with pleiotropic biological and pharmacological activities on central and peripheral nervous systems (Chiechio et al., 2017). ALCAR has a key role in neuronal metabolism (i.e., β-oxidation, glycogen production, glucose utilization, and ammonia cycle), growth, plasticity, and regeneration; ALCAR is actively taken up by the brain and modulates the release of aminergic neurotransmitters and the biosynthesis and release of glutamate (Kuratsune et al., 2002; Tolu et al., 2002; Tanaka et al., 2003; Freo et al., 2009; Smeland et al., 2012; Chiechio et al., 2017; Wang et al., 2014; Burks et al., 2019). Exogenous ALCAR may increase neurogenesis in prefrontal–limbic areas via a selective upregulation of mGluR2 receptors, by acting as histone acetylator on transcription factors of the nuclear factor (NF)-kappa B family (Chiechio et al., 2006; Nasca et al., 2013).

In experiment animals, ALCAR is neuroprotective against hypoxia, nerve and spinal cord injury, and neurotoxins such as amphetamines and 1-methyl-4-phenyl-1,2,3,6-tetrahydropyridine (Chiechio et al., 2017; Burks et al., 2019). In humans, low plasma levels of ALCAR have been associated with an increased vulnerability to chronic pain and depression. Specifically, circulating ALCAR was found to be reduced in patients with severe osteoarthritis pain that did not improve after a total joint replacement (Costello et al., 2020). ALCAR was found to be reduced also in the plasma and the brain of patients with chronic fatigue or major depressive disorder and, more markedly, when depression was severe, treatment resistant or associated with history of childhood trauma or neglect (Kuratsune et al., 2002; Nasca et al., 2018; Post, 2018; Pu et al., 2020).

Given its excellent, long-term safety profile, ALCAR has been studied in several neurological and psychiatric conditions, confirming a strong antineuropathic activity in toxic and traumatic painful neuropathies (Onofrj et al., 2013; Chiechio et al., 2017). Specifically, ALCAR improved the function of peripheral nerves by reducing sensory neuronal loss and by enhancing nerve regeneration and conduction velocity (Onofrj et al., 2013; Cruccu et al., 2017). Administration of ALCAR has consistently shown good tolerability and efficacy in dysthymic disorder and on depressive symptoms associated with fibromyalgia or minimal hepatic encephalopathy (Wang et al., 2014).

### Ketamine

Ketamine is an arylcycloalkylamine structurally analogue to phencyclidine (PCP, *angel dust*), an approved anesthetic and the prototypical, non-competitive *N*-methyl-D-aspartate receptor-glutamate (NMDA) antagonist.

In addition to NMDA antagonism, however, KET has a myriad of effects on neurotransmitters which include the following: increase of synaptic concentrations of acetylcholine in the spinal cortex, hippocampus, and neocortices; increase of glutamate, serotonin, and noradrenaline in the prefrontal cortex; increase of dopamine in the basal ganglia and neocortices; activation of synaptogenic α-amino-3-hydroxy-5-methyl-4-isoxazolepropionic acid (AMPA) receptors; and activation of synaptogenic intracellular signaling, including mammalian target of rapamycin complex (TORC1) (Freo and Ori, 2002b, 2003; Cohen et al., 2018). Behaviorally, KET has almost unique, dose-dependent effects on the central nervous system. Different from most general anesthetics, at high, anesthetic doses, KET and few other congeners produce a “dissociative anesthesia” during which patients may appear awake and maintain spontaneous eye movements and respiratory drive although they are relatively insensitive to sensory stimulation (Cohen et al., 2018). At lower subanesthetic doses, KET promotes arousal from anesthesia and has strong antidepressant and analgesic activities (Hambrecht-Wiedbusch et al., 2017; Cohen et al., 2018). After a low intravenous dose of KET (0.5 mg/kg), most patients with a major depressive disorder reported a very fast, within hours, improvement of depressive symptoms that lasted for 7–10 days (Cohen et al., 2018). The antidepressant effects of KET were replicated in patients with treatment-resistant or bipolar depression, suggesting that KET may be effective on a wider range of depressed patients (Romeo et al., 2015).

In addition, low-dose KET has been beneficial on large numbers of patients suffering from oncological and non-oncological pain (Cohen et al., 2018; Orhurhu et al., 2019). The consensus guidelines recently elaborated by the American Society of Regional Anesthesia and Pain Medicine, the American Academy of Pain Medicine, and the American Society of Anesthesiologists support the use of KET for chronic pain, but with different degrees of evidence for different conditions and dose ranges (Cohen et al., 2018). Adverse events of KET were similar to those of placebo, with higher dosages and more frequent infusions being associated with greater risks (Cohen et al., 2018). However, not all authors agree and dismiss KET adverse events as anecdotal. In clinical practice, using KET for chronic pain or depression is still limited by intravenous administration and its potential neurotoxic and toxic effects (Jevtovic-Todorovic et al., 2001; Liao et al., 2010; Orhurhu et al., 2020). However, intranasal and oral administration as well as the discovery of antidepressant activity and lower psychotomimetic effect of isomers and metabolites of KET may foster a wider and longer-term clinical use of KET in the future (Zanos et al., 2016). Because S(+)KET has higher affinity for NMDA receptors than R(−)KET, S(+)KET has been developed and later approved by the Food and Drug Administration as a clinical antidepressant (Hashimoto, 2019). However, in animal models of depression, R(−)KET has shown longer-lasting antidepressant effects and lesser adverse effects than S(+)KET and is currently being investigated as a promising antidepressant (Hashimoto, 2019).

## Cerebral Metabolism Studies

The regional cerebral metabolic rates for glucose (rCMRglc) were measured using the quantitative autoradiographic [^14^C]2-deoxy-D-glucose technique in groups of five to seven male, Fischer-344, conscious rats at 30 min after IV administration of saline or ALCAR 250–750 mg/kg and at 20 min after IV saline or KET 20 mg/kg (Ori et al., 2002; Freo and Ori, 2004). The [^14^C]2-deoxy-D-glucose procedure has been detailed previously (Freo and Ori, 2002a, 2004, 2009).

**FIGURE 1:**
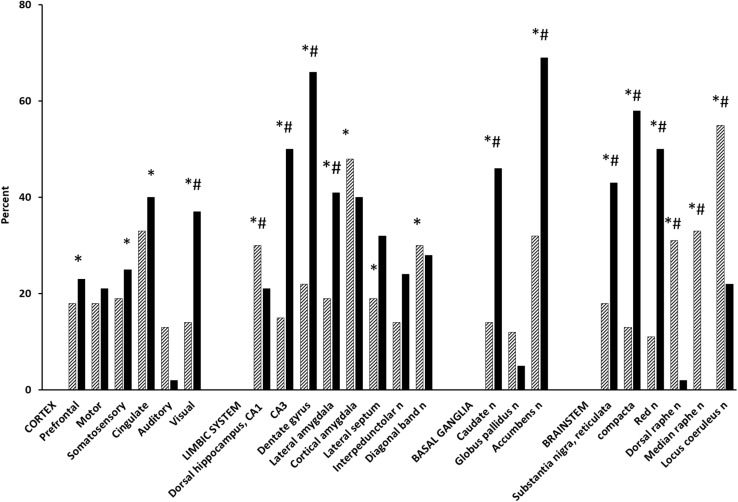
Effect of ALCAR and KET on rCMRglc. Bars are mean rCMRglc differences (percent) from saline controls in groups of 5–7 Fischer-344, male rats at 30 min after IV administration of ALCAR 500 mg (hatched) and 20 min after IV KET 20 mg (solid). ALCAR difference from saline control: ^∗^*P* < 0.05; ALCAR difference from KET: ^#^*P* < 0.05.

Acetyl-L-carnitine dose-dependently increased rCMRglc in the prefrontal, cingulate, and somatosensory cortices, in the cortical amygdala and in the accumbens, diagonal band, dorsal raphe, and locus coeruleus nuclei (ANOVA and unpaired *t*-test, *P* < 0.05) (Figure 1; Ori et al., 2002). Acetate and carnitine alone had no effect on cerebral metabolism, indicating that rCMRglc increases by ALCAR are independent from its effects on mitochondrial metabolism (Ori et al., 2002). KET [*S*,*R*(±)-ketamine] increased rCMRglc similarly to ALCAR in cortical areas, to a lesser extent in serotoninergic raphe nuclei and to a much greater extent in hippocampal regions and dopaminergic nuclei (average percentage increase 32.2 ± 11.4 vs. 20.0 ± 23.0; *P* < 0.01) (Freo and Ori, 2004). Using functional magnetic resonance imaging in paralyzed, mechanically ventilated rats, Masaki and coworkers reported that *S*,*R*(±)KET 10 mg/kg and S(+)KET 10 mg/kg increased the regional cerebral blood flow signal in the basal ganglia and cortical regions in a similar fashion to that in MK801 (Masaki et al., 2019); in contrast, the same dose of R(−)KET produced no noticeable behavioral change and a widespread decrease of regional cerebral blood flow (Freo and Ori, 2004; Masaki et al., 2019). In GluN2D-knockout mice, KET failed to increase the [^14^C]-2-deoxy-D-glucose uptake as well as the cortical gamma-band power, suggesting that prefrontal cortical activations are mediated by mGluR2 receptors (Sapkota et al., 2016). Furthermore, in humans, KET increased the [^18^F]-fluorodeoxy-D-glucose uptake in the prefrontal cortex in a correlative fashion to post-treatment antidepressant effects (Li et al., 2016). Finally, the antidepressant effects of KET continuing beyond its pharmacokinetic half-life was associated with persistent activation of the frontal supplementary motor and cingulate cortices (Chen et al., 2018).

While the rCMRglc effects of KET were similar to those of other non-competitive NMDA antagonists, they differ markedly from the small rCMRglc changes induced by competitive NMDA receptor antagonists (i.e., AP7, CGP39551, CPP, and CGS19755), which have demonstrated relatively modest antidepressant effects and to actually counteract KET-induced dopamine activations (French, 1992; Sharkey et al., 1996; Iadarola et al., 2015). Pre-clinical and (Freo and Ori, 2002a,b) human studies have reported abnormalities of glutamatergic systems in depression (Jiménez-Sánchez et al., 2016); conversely, enhancement of glutamate neurotransmission in the prefrontal cortex is considered necessary and sufficient for the antidepressant properties of glutamate drugs (Fukumoto et al., 2016; Highland et al., 2019). However, ALCAR and KET differ in their mechanisms of action and can lead to prefrontal activation in two different manners: ALCAR may do so directly via upregulation of mGluR2 receptors, KET may act indirectly via an NMDA antagonism on GABA inhibitory neurons, and the subsequent disinhibition of pyramidal cortical neurons (Moghaddam et al., 1997). Enhanced AMPA/glutamate transmission by KET stimulates, in turn, release of adrenaline and serotonin, which may contribute to KET antidepressant effects (Fukumoto et al., 2016; Jiménez-Sánchez et al., 2016).

Compared to ALCAR, KET determined greater rCMRglc increases in dopaminergic nuclei (i.e., accumbens and substantia nigra, pars reticulata and compacta, nuclei: 233, 138, and 346%, respectively, *P* < 0.01) and in hippocampal areas (i.e., dorsal CA_3_ and dentate gyrus: 233 and 200%, respectively, *P* < 0.01), which are among the largest metabolic activations ever reported (Sharkey et al., 1996). However, they are consistent with the marked increases KET elicits also on electrical activity in the ventral tegmental area, extracellular concentrations of dopamine in the nucleus accumbens and prefrontal cortex, and hyperlocomotion by the dopamine D_2__/__3_ receptor agonist quinpirole (Witkin et al., 2016). KET dopaminergic activations were prevented by dopaminergic neuroleptics and an AMPA receptor antagonist, indicating AMPA-dependent effects (Duncan et al., 2003; Witkin et al., 2016). While the role of dopaminergic and glutamatergic activations in KET antidepressant actions remains questionable, the large rCMRglc increase KET induces in mesolimbic areas likely reflects dopamine “surges” that mediate natural and drug rewards and, possibly, the abuse liability of KET (Kokkinou et al., 2018; Volkow et al., 2019). In contrast, ALCAR increases much less dopamine release and rCMRglc; although it is faster than conventional selective serotonin and/or norepinephrine reuptake inhibitor and tricyclic antidepressants, ALCAR is a less potent and slower antidepressant than KET and is devoid of abuse risk (Tolu et al., 2002; Romeo et al., 2015; Chiechio et al., 2017).

In experiment animals, KET has potential neuroprotective properties in stroke, neurotrauma, subarachnoid hemorrhage, and status epilepticus; however, KET has been reported to cause also some worrisome neurotoxic damage, which, interestingly, can be counteracted by ALCAR (Jevtovic-Todorovic et al., 2001; Liao et al., 2010; Robinson et al., 2016; Bell, 2017; Orhurhu et al., 2020).

Acetyl-L-carnitine increased rCMRglc to a similar extent in most brain areas in young and aged rats and to a larger extent in the limbic regions of aged rats (Freo et al., 2009). Following its chronic administration, ALCAR determined larger rCMRglc increases in hippocampal areas, which are crucial to attention and memory functions (Freo et al., 2009). Because ALCAR is endowed with cholinomimetic properties, its positive effects on attention and memory were ascribed to ALCAR cholinergic agonism (Battistin et al., 1989; Jeong et al., 2017). However, as the limbic regions are electrically and metabolically hyporesponsive to acute cholinergic muscarinic stimulation and to chronic cholinergic treatment, a non-cholinergic mechanism for memory-enhancing effects of ALCAR is likely (Freo et al., 2009). During aging, the glutamate neurotransmission undergoes complex changes within the hippocampus, which include increases of glutamate-induced phosphoinositol hydrolysis, of densities of glutamate receptors (i.e., mGluR2, mGluR3, and mGluR5) and of their mRNAs, all of which have been interpreted as compensatory for age-related alteration of glutamate neurotransmission (Griego and Galván, 2020). The aging cognitive decline has been associated with weakened synaptic strength in prefrontal and hippocampal regions. Interestingly, riluzole, a glutamate release inhibitor and glutamate antagonist, was shown to increase glutamatergic activity in the hippocampus, preventing thus cognitive decline during aging (Pereira et al., 2014). Hence, ALCAR may also have a positive effect on cognitive functions by activating the hippocampal glutamatergic mechanisms.

## Clinical Studies

### Effects of ALCAR on Chronic Pain

Chronic pain and depression often coexist, requiring frequent or continuous treatments (Freo et al., 2019a). Multi-pathologies and multi-therapies make it challenging especially in the elderly and frail population. In comorbid chronic pain and depression, ALCAR may be useful because of its analgesic and antidepressant properties and high long-term tolerability (Chiechio et al., 2017).

We investigated the effects of ALCAR in painful neuropathies and radiculopathies that were unresponsive or poorly responsive (i.e., ≤30% pain relief) to previous therapies in 28 patients (17 females and 11 males; age 66.4 ± 10.1 years; pain duration 16 ± 21 months) (Freo et al., 2019a). The primary outcome was pain intensity after a 4-month treatment with ALCAR 500 mg BID that was given initially IM for an average of 57 ± 9 days and then PO. Patients were assessed for the 24-h average pain with a 0–10 numerical rating scale (NRS) (pain rating, 0 = no pain, 1–3 = mild, 4–6 = moderate, and 7–10 = severe pain), for neuropathic pain symptoms with the painDETECT questionnaire [<12 = negative (nociceptive pain), 13–18 = uncertain (mixed pain), ≥19 = positive (neuropathic pain)], for depressive symptoms with the Hospital Anxiety and Depression Scale (HADS) (<7 = no depression, 8–10 = mild, 11–15 = moderate, and 16–21 = severe depression), and for quality of life with the 12-item Short Form Health Survey (SF-12), physical and mental components (Freo et al., 2019a,b).

**FIGURE 2:**
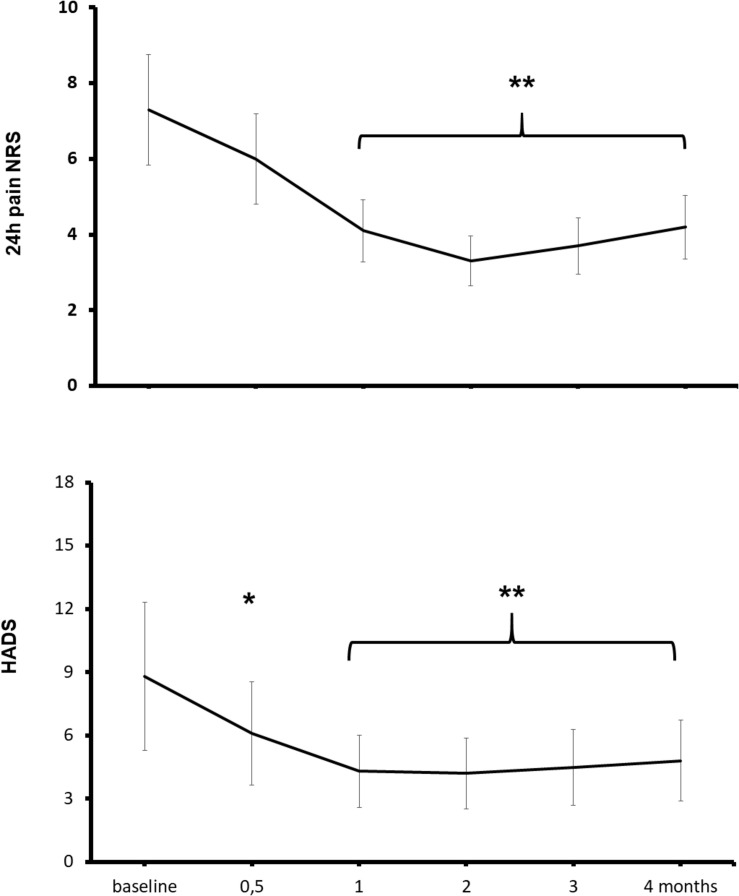
Effects of ALCAR on chronic pain and depression. Points are means ± standard deviation of 24-h average pain NRS scores (above) and depression HADS scores (below) from pre-treatment baseline to month 4 treatment in 28 patients receiving ALCAR 500 mg BID IM/PO for chronic neuropathy or radiculopathy pain. Significantly different from baseline: *P < 0.05; **P < 0.01.

At baseline, all patients reported a moderate-to-severe, 24-h average pain (NRS ≥ 4/10), 60% of patients reported symptoms of a mild-to-moderate depression (HADS ≥ 8), and 57% had a positive painDETECT score (≥12) for neuropathic pain (Freo et al., 2019a). The 4-month treatment with ALCAR was associated with a reduction of pain and depression (Figure 2). Pain intensity significantly improved from baseline to month 1 of treatment (pain NRS from 7.4 ± 1.5 to 5.6 ± 1.7; means ± standard deviation, Kruskal–Wallis and Wilcoxon’s test, *P* < 0.01) and depressive symptoms improved already at week 2 of treatment (HADS scores from 8.8 ± 4.4 to 6.1 ± 3.4, *P* < 0.01) (Figure 2; Freo et al., 2019a). Compared to baseline, at month 4 outcome, a moderate (30–49%) pain improvement was observed in 11 patients and a substantial (≥50%) improvement in 8 patients. The painDETECT score for neuropathic pain decreased from baseline to month 4 outcome from 12.6 ± 6.0 to 5.0 ± 0.9 (*P* < 0.01); the SF-12 mental component increased from 44.1 ± 4.5 to 53.5 ± 5.1 (*P* < 0.01) and the SF-12 physical component from 35.3 ± 3.5 to 39.4 ± 5.2 (not significant). Five patients discontinued treatment because of lack of efficacy or unwillingness to continue treatment; no adverse effect was recorded (Freo et al., 2019a).

Reportedly, ALCAR improved pain and nerve function in experimental and clinical neuropathies of different etiologies with therapeutic effects being ascribed mainly to ALCAR neuroprotective and neuroregenerative properties (Li et al., 2015). However, although in peripheral neuropathies, depression is common and noradrenaline–serotonin reuptake inhibitor and tricyclic antidepressants are first-line treatments, depressive symptoms are not always measured. Pain and depression have a biunivocal relation with worsening or improvement in one variable predicting subsequent changes in severity of the other (Kroenke et al., 2011; D’Amato et al., 2016). As such, rapid-acting antidepressant and analgesic drugs have been a major breakthrough (Chiechio et al., 2017). In our patients, ALCAR improved depressive symptoms earlier than pain symptoms, suggesting that the antidepressant activity of ALCAR may anticipate and contribute to its analgesic properties.

Chronic neuropathic pain and depression are age-dependent, highly comorbid disorders that complicate courses and outcomes (Dworkin et al., 2003; Brouwer et al., 2015; Hanewinckel et al., 2016; Freo et al., 2019b). Therapeutic responses are often poor and limited by concurrent therapies. The elderly population is at increased risk for adverse events from antidepressants and anticonvulsants that may worsen stability, balance, and cognition (Dworkin et al., 2003; Brouwer et al., 2015; Hanewinckel et al., 2016). Because of its high tolerability and the positive effect it has on pain, depression, and cognition, ALCAR should be in the therapeutic armamentarium for treating comorbid pain and depression, especially in the elderly population.

### Effects of KET on Post-operative Pain

Almost unique among general anesthetics, the NMDA antagonist KET has anesthetic properties with low cardiovascular and respiratory depression (Cohen et al., 2018). KET is also clinically attractive because it has strong analgesic and antidepressant activities and may prevent central sensitization and hyperalgesia (Freo and Ori, 2003; Ori et al., 2003; García-Henares et al., 2018). Hence, KET is of interest for patients prone to anesthesia-induced respiratory impairment or suffering from chronic pain and/or depression or, more, for patients presenting with all these clinical features such as the morbidly obese (Freo and Ori, 2003; Ori et al., 2003; Carron et al., 2012; García-Henares et al., 2018).

In fact, overweight and obesity are frequently associated with an obstructive apnea syndrome and/or to a depressive disorder that places patients at risk, respectively, of post-operative critical events and of post-operative complications and prolonged stay (Luppino et al., 2010; Ghoneim and O’Hara, 2016; Subramani et al., 2017; Nijland et al., 2020). Obesity is associated with higher rates of chronic pain and higher scores of post-operative pain, which are both challenging to treat in this patient population (Belcaid and Eipe, 2019; Mills et al., 2019). As opioids may induce ventilatory impairment, multimodal opioid- and muscle relaxant-sparing techniques are being investigated to improve the safety of analgesia in obese patients; in this regard, KET may present specific advantages (Ori et al., 2003; Dalsasso et al., 2005; Freo et al., 2011; Carron et al., 2012; García-Henares et al., 2018; Aronsohn et al., 2019).

The effects of KET as the main anesthetic agent were determined in 500 patients (172 males and 328 females, ASA I–II, age 53.9 ± 12.2 years, weight 76.1 ± 22.5 kg) undergoing an opioid-free anesthesia for day surgery including breast surgery, laparoscopy, superficial excision of minor lesions, thoracoscopy, appendectomy, and proctology (Dalsasso et al., 2005). At induction, patients received IV midazolam 0.03–0.05 mg/kg, clonidine 150 μg, and KET 0.4 mg/kg; the latter was repeated as needed during surgery (mean total dose 0.6 ± 0.2 mg/kg). Anesthesia was maintained with nitrous oxide and sevoflurane. Seventy-four percent of patients were eligible to discharge from the operating theater by 30 min, and all patients were dischargeable by 1 h. Patients did not complain of hallucinations, while presenting a high rate of satisfaction at the Iowa Satisfaction with Anesthesia Scale (Dalsasso et al., 2005).

The post-operative effects of KET were assessed in 41 obese patients (26 females and 15 males; age 42.7 ± 10.7 years; body mass index 44.5 ± 7.2) undergoing laparoscopic gastric banding or sleeve gastrectomy with the primary outcomes being post-operative pain and depression (Freo, 2020). All patients were pre-medicated with midazolam and induced with IV propofol 1.5 mg/kg and fentanyl 1–2 μg/kg and maintained with sevoflurane 1–2%; patients were randomized to receive at induction either saline or KET [S, R(±)ketamine 0.5 mg/kg by ideal body weight]. Baseline demographic features (i.e., age, education, body mass index, comorbidities, and medical therapies), times of anesthesia and surgery, and average propofol dosages were similar between groups; average fentanyl dosage was higher in the saline control than in the KET group (i.e., 341 ± 109 and 192 ± 67 mg, *P* < 0.01) (Freo, 2020).

**FIGURE 3:**
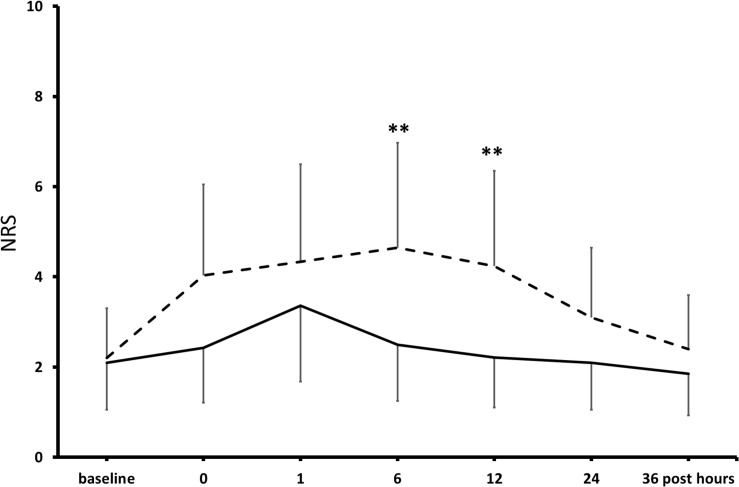
Effects of KET on post-operative pain in obese bariatric patients. Points are means ± standard deviations of NRS scores of pain in the first 36 h after bariatric surgery in 41 obese patients who had received either saline (broken line) or KET 0.5 mg/kg (continuous line) at induction of anesthesia. Significantly different from saline controls: **P < 0.01.

**FIGURE 4:**
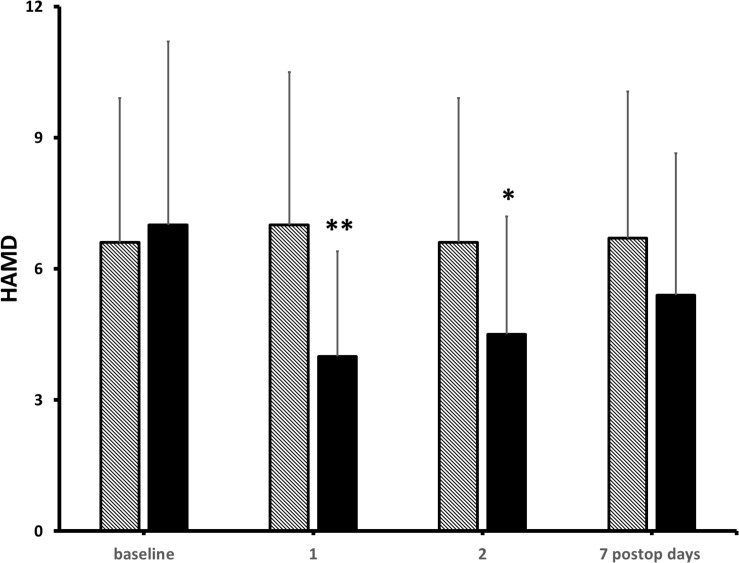
Effects of KET on depression in obese bariatric patients. Columns are means ± standard deviations of HAMD scores from baseline to post-operative day 7 in 41 bariatric patients who had received either saline (hatched columns) or KET 0.5 mg/kg (solid columns) at induction of anesthesia. Significantly different from saline controls: ^∗^*P* < 0.05; ^∗∗^*P* < 0.01.

At pre-operative baseline, in the control and the KET groups, seven and eight patients reported moderate-to-severe pain (NRS ≥ 4/10), and 9 and 10 patients presented mild-to-moderate depressive symptoms in the Hamilton Depression Rating Scaling (HAMD), respectively (Freo, 2020). Pain scores were significantly lower at post-operative hours 6 and 12 in KET-treated patients than in controls and then subsided in both groups (Figure 3). Pain improvement was less in non-depressed than depressed patients (mean intergroup difference at post-operative day 1, −33 and −51%, *P* < 0.05) (Freo, 2020). HAMD scores were significantly lower in the KET-pre-treated patients at the post-operative days 1 and 3 (HAMD scores in saline and KET groups at baseline: 6.7 ± 5.7 and 7.1 ± 5.7; at post-operative day 1: 6.7 ± 5.7 and 3.4 ± 2.6; at post-operative day 3: 7.0 ± 5.6 and 3.7 ± 2.9; Friedman’s and Mann–Whitney *U* tests, *P* < 0.01) (Figure 4; Freo, 2020).

A recent Cochrane meta-analysis review concluded that perioperative intravenous KET reduces post-operative pain and nausea and analgesic consumption (Brinck et al., 2017); however, not all studies are consistent with these findings. In a recent randomized controlled study (RCT) on 100 obese patients, of whom 22 with history of depression and 13 with history of chronic pain, undergoing laparoscopic gastric bypass or gastrectomy, post-operative infusion of KET (0.4 mg/kg, ideal body weight) was not superior to placebo on post-operative pain and mood assessed with a pain Visual Analogue Scale, the Beck Depression Inventory and the Montgomery–Asberg Depression Rating Scale; KET, however, improved the affective and the total score of the short-form McGill Pain Questionnaire starting on post-operative day 2 (Wang et al., 2019). In the PODCAST multicenter RCT on 672 older adults (i.e., >60 years) undergoing cardiac and non-cardiac surgery, pre-incisional KET (0.5 or 1 mg/kg) did not decrease post-operative pain, delirium, or depressed mood (Avidan et al., 2017).

Most negative studies focused on post-operative pain. Analgesic effects of KET are considered use dependent: the worst the pain, the more efficient KET will be as analgesic (Robu and Lavand’homme, 2019). The same may hold true for its antidepressant effect. KET has a plasma half-life of 2.3 ± 0.5 h with a duration of action of IV bolus of 5–10 min (Cohen et al., 2018). KET has a rapid and potent antidepressant effect that peaks at 24–48 h after administration and could have a larger impact on patients with mood disorders (Cohen et al., 2018). Consistently, Kudoh et al. (2002) reported that KET significantly improved mood and pain on post-operative day 1 in depressed patients undergoing orthopedic surgery. In a second double-blind RCT, KET 0.5 mg/kg IV bolus followed by a 30 min infusion of 0.25 mg/kg/h increased mood and serum brain-derived neurotrophic factor, which is a marker of major depressive disorders and treatment response (Jiang et al., 2016). It is therefore possible that the analgesic effect of KET is contributed at least in part by its antidepressant activities. In our sample, 15 patients (37%) had at least a mild-to-moderate depression, and nine patients were on chronic antidepressant therapy; large-cohort studies indicated that the prevalence of subclinical depression ranges from 1 to 17% (Heo et al., 2006), which suggests that obese patients with mood disorders may especially benefit from KET treatment.

Ketamine is endowed with a peculiar profile with multiple pharmacological activities that may result from different underlying mechanisms. Other NMDA antagonists (i.e., MK801 and memantine) do not have the same anesthetic, analgesic, and antidepressant effects of KET, leaving open the question of its mechanisms of action (Kelland et al., 1993; Gould et al., 2019; Robu and Lavand’homme, 2019). Besides the glutamate system, KET interacts with several other neurotransmitter systems (Jelen et al., 2020). For example, the administration of the insulin growth factor small-interfering RNA blocks KET antidepressant effects in the mouse learned helplessness model of depression (Grieco et al., 2016). In rodents, activation of AMPA receptors by the KET metabolite (2R,6R)-hydroxynorketamine has initially been thought essential to replicate KET antidepressant effects while administration of 2,3-dihydroxy-6-nitro-7-sulfamoyl-benzo[f]quinoxaline-2,3-dione (NBQX), an AMPA receptor antagonist, blocks KET effects (Zanos et al., 2016). However, in subsequent investigations in rodent models of depression, (2R,6R)-hydroxynorketamine did not exhibit antidepressant-like effects and increased aggressive behavior (Yang et al., 2019). The brain-derived neurotrophic factor (BDNF) and its receptor, tyrosine kinase receptor B (TrkB), are essential and common mechanisms for the antidepressant effects of both the parent molecules of ketamines [i.e., S, R(±)ketamine, S(+)ketamine, and R(−)ketamine] and their active metabolites [i.e., (2R,6R)-hydroxynorketamine and of S(+)norketamine] (Yang et al., 2019). In patients with treatment-resistant depression, naltrexone 50 mg blocked the antidepressant but not the dissociative effects of KET, suggesting that the opioid system may also be required for KET antidepressant activities (Williams et al., 2018). Other works, however, have shown that naltrexone pre-treatment did not affect the antidepressant activities of KET in depressed individuals (Marton et al., 2019; Yoon et al., 2019; Zhang and Hashimoto, 2019).

## ALCAR and KET as Glutamate Drugs for Pain and Depression

The excitatory actions of glutamate in the central nervous system have been recognized in the early 1950s (Curtis and Watkins, 1960). Since the discovery of neurotoxic effects of massive glutamate release, glutamate antagonists have been trialed in massive neuronal damage (i.e., stroke and brain and spinal cord trauma), with disappointing results (Ikonomidou and Turski, 2002). Later, it was shown that glutamate is actually essential to physiological neuroplasticity underlying learning and memory, as well recovery from brain stroke or trauma (Ikonomidou and Turski, 2002).

The report of the fast antidepressant effects of KET, a non-selective glutamate agent, fostered interest in the role of glutamate in specific neurological functions (Henter et al., 2017; Pereira and Goudet, 2019). Reportedly, glutamate is involved in maladaptive neuroplastic processes contributing to generation and maintenance of pain and mood disorders (Riggs and Gould, 2021). Frontal areas are critical for top-down cognitive modulation of pain and pain-related emotions (Thompson and Neugebauer, 2019). A lower level of frontal activity has been linked to higher pain and low mood, and conversely, an increased frontal activity has been linked to analgesic and antidepressant effects (Thompson and Neugebauer, 2019). Furthermore, glutamate concentrations are reduced in frontal areas in experimental and clinical pain (Thompson and Neugebauer, 2019), and a frontal glutamatergic dysfunction has been implicated in depression as well (Moriguchi et al., 2019). Therefore, modulation of glutamate neurotransmission is a current research target for pain and depression (Pereira and Goudet, 2019; Riggs and Gould, 2021).

The number of glutamate-receptor-selective agents has been fast growing, but the clinical safety still remains an issue for most new agents (Henter et al., 2017; Pereira and Goudet, 2019). Also, more generally, development of non-glutamate drugs for pain and depression has been plagued by failures in advanced human trials. As a consequence, older glutamate drugs are being reassessed.

Acetyl-L-carnitine and KET are non-selective, clinical glutamate modulators which have been shown to improve pain and depressed mood in different experimental and clinical settings; they share the common properties of increasing brain glutamate concentration and neurotransmission and of activating rCMRglc in frontal areas and subcortical aminergic nuclei (Toth et al., 1993; Moghaddam et al., 1997; Fukumoto et al., 2016; Jiménez-Sánchez et al., 2016). These findings are consistent with frontal and brainstem activations occurring during opioid, placebo (Petrovic et al., 2002), and other types of analgesia and in individuals resilient to depression (Ong et al., 2019; Fischer et al., 2021). Prefrontal and frontal cortices are pivotal components of the “pain matrix” and of the fronto-limbic, frontostriatal, and default-mode networks that regulate pain perception, emotionally driven behaviors, and attention allocation (Li et al., 2018; Ong et al., 2019). A prefrontal dysfunction/hypofunction has been associated with abnormal pain processing and with loss of pleasure, motivational energy, cognitive abilities, and speed. In contrast, increased frontal activation and/or normalization of abnormal connectivity have been associated with improvement of pain and of sad mood (Li et al., 2018; Ong et al., 2019). ALCAR and KET also activated brainstem nuclei (locus coeruleus, diagonal band, and raphe nuclei) which send aminergic projections to cortical areas and spinal dorsal horns that are involved in pain and mood control (Freo et al., 2010; Khan and Stroman, 2015).

Altogether, the findings indicate that non-selective, clinical glutamate modulators such as ALCAR and KET can still provide therapeutic benefits and generate hypotheses on glutamate drug actions in neuropsychiatric conditions.

## Author Contributions

UF and GZ contributed to the conception and design of the study and wrote sections of the manuscript. UF wrote the first draft of the manuscript. All authors revised, read, and approved the submitted version.

## Conflict of Interest

The authors declare that the research was conducted in the absence of any commercial or financial relationships that could be construed as a potential conflict of interest.
